# Methods to isolate a large amount of generative cells, sperm cells and vegetative nuclei from tomato pollen for “omics” analysis

**DOI:** 10.3389/fpls.2015.00391

**Published:** 2015-06-02

**Authors:** Yunlong Lu, Liqin Wei, Tai Wang

**Affiliations:** ^1^Key Laboratory of Plant Molecular Physiology, Institute of Botany, Chinese Academy of SciencesBeijing, China; ^2^University of Chinese Academy of SciencesBeijing, China

**Keywords:** *Solanum lycopersicum*, generative cell, sperm cell, vegetative nuclei, isolation, Percoll density gradient centrifugation

## Abstract

The development of sperm cells (SCs) from microspores involves a set of finely regulated molecular and cellular events and the coordination of these events. The mechanisms underlying these events and their interconnections remain a major challenge. Systems analysis of genome-wide molecular networks and functional modules with high-throughput “omics” approaches is crucial for understanding the mechanisms; however, this study is hindered because of the difficulty in isolating a large amount of cells of different types, especially generative cells (GCs), from the pollen. Here, we optimized the conditions of tomato pollen germination and pollen tube growth to allow for long-term growth of pollen tubes *in vitro* with SCs generated in the tube. Using this culture system, we developed methods for isolating GCs, SCs and vegetative cell nuclei (VN) from just-germinated tomato pollen grains and growing pollen tubes and their purification by Percoll density gradient centrifugation. The purity and viability of isolated GCs and SCs were confirmed by microscopy examination and fluorescein diacetate staining, respectively, and the integrity of VN was confirmed by propidium iodide staining. We could obtain about 1.5 million GCs and 2.0 million SCs each from 180 mg initiated pollen grains, and 10 million VN from 270 mg initiated pollen grains germinated *in vitro* in each experiment. These methods provide the necessary preconditions for systematic biology studies of SC development and differentiation in higher plants.

## Introduction

During the development of sperm cells (SCs, male gamete) from microspores in higher plants, the microspore generated from diploid microsporocytes via meiosis first undergoes asymmetric mitosis to produce a larger vegetative cell (VC) and a smaller generative cell (GC) embedded in the VC. Thereafter, the VC exits the cell cycle and has potential to generate a polarly growing pollen tube; the GC enters further mitosis to produce two SCs for double fertilization (McCormick, 1993; Twell et al., 1998; Twell, 2011). Depending on the plant species, GC mitosis occurs before anthesis or in growing pollen tubes; therefore, released mature pollen at anthesis is tricellular in some species such as *Oryza sativa*, *Zea mays*, and *Arabidopsis thaliana* (Berger and Twell, 2011) or bicellular in other species such as *Lilium brownii* and *Solanum lycopersicum*. This development process involves a set of fine-tuned molecular and cellular events and the coordination of these events, such as cell cycle regulation, cell differentiation and fate determination, genome stability, and epigenetic reprogramming. Although genetic studies have functionally identified many important genes involved in plant SC development, such as *DUO1*, *DUO3*, *DAZ1*, and *DAZ2* (Borg et al., 2009, 2011, 2014; Brownfield et al., 2009a,b; Twell, 2011), the mechanisms underlying these events and their interconnections remain a major challenge for plant science. Systematic “omics” studies of the development process are essential for understanding the mechanisms.

“Omics” studies of pollen from several plants including *Arabidopsis* and rice have provided insights into the molecular mechanisms of pollen development (Rutley and Twell, 2015). During postmeiotic development from microspores, pollen express a set of specific transcripts; the total number of transcripts expressed is decreased, but the proportion of pollen-specific or preferential transcripts is increased (Honys and Twell, 2004; Wang et al., 2008; Wei et al., 2010). The composition and expression profile of miRNAs expressed in developing pollen differs from those in sporophytes, and novel and non-conserved known miRNAs are the main contributors to the difference (Wei et al., 2011). In pollen, small RNA displays cell-specific activity: working by translational repression in the SC, and by cleavage-induced mRNA turnover in the VC (Grant-Downton et al., 2013). The small RNA from the VC are strongly implicated in gene silencing in SCs (Slotkin et al., 2009; Grant-Downton et al., 2013). This indicates reprogramming of gene expression during pollen development and the importance of epigenetic signals in this reprogramming. In addition, proteomics and metabolomics studies have revealed the importance of presynthesized proteins during pollen maturation in pollen function (Holmes-Davis et al., 2005; Dai et al., 2006), and difference in proteomes and metabolitic pathways between mature and germinated pollen (Dai et al., 2007; Obermeyer et al., 2013). These studies also revealed many important candidate genes for further understanding the molecular control of pollen development by functionally dissecting these candidates.

Recent studies have isolated SCs from tricellular pollen of rice and *Arabidopsis* and analyzed the transcriptome of SCs (Borges et al., 2008; Russell et al., 2012). The transcriptome of the SC was significantly different from that of the pollen grain, which is consistent with the SC being only a little part of the pollen grain that is mainly represented by the VC. SC-preferential transcripts showed a prominent functional skew toward epigenetic regulation, DNA repair, and cell cycles (Borges et al., 2008; Russell et al., 2012). Small RNA-mediated DNA methylation in SCs is associated with epigenetic inheritance, transposon silencing and paternal imprinting (Borges et al., 2008; Calarco et al., 2012). Further systematic “omics” analysis of molecular programs for SC development from its precursors, the GC and microspore, is essential to understand the mechanism of SC development. To achieve this goal, we need to establish a condition to isolate GCs and SCs from the pollen of a species. Because the GC occurs at a short time window *in vivo* and develops asynchronously in different flowers in rice and *Arabidopsis*, isolating a large amount of GCs at high purity from developing pollen of these species for “omics” analysis is difficult.

Tomato is another model plant to study pollen development (Twell et al., 1990, 1991; Muschietti et al., 1994; Filichkin et al., 2004) and can be an excellent model to achieve the above target because (1) its genome has been sequenced (Sato et al., 2012) and (2) its mature pollen is bicellular. This feature of pollen indicates the possibility to isolate GCs from pollen grains or just-germinated pollen grains (JGPGs) and to isolate SCs from pollen tubes with SCs formed from GCs via mitosis.

In this study, we optimized the conditions of pollen germination and pollen tube growth to allow for long-term growth of pollen tubes *in vitro* with SCs generated in the tube. Using this culture system, we developed efficient protocols to isolate a large amount of GCs, SCs, and vegetative cell nuclei (VN) at high purity to satisfy the demands of “omics” study.

## Materials and Methods

### Plants Growth and Pollen Collection

Tomato (*S. lycopersicum*) plants (Heinz 1706) were grown in the greenhouse under long-day conditions (14 h light/10 h dark) at 25 ∼ 35°C. During anthesis, anthers from opened flowers were collected and dried for 10 h at 28°C in an electrothermal drying closet, then placed into a colander (85 mm × 50 mm) with mesh at 63-μm-pore size; mature pollen was released and collected by shaking the colander vigorously. Pollen grains were used immediately or stored in a 1.5 mL tube with 5∼10 particles of Silica gel Rubin (Sigma, 85815) at −20°C.

### Pollen Germination *In Vitro* and Morphologic Observation

Mature pollen grains (60 mg) were pre-hydrated in a Petri dish (60 mm × 15 mm), which was covered with gauze and then placed in a large Petri dish (150 mm × 25 mm) with 50 mL saturated Na_2_HPO_4_ at 25°C for 4∼8 h. This device only permitted gauze contact this solution, and prohibited pollen grains contact the gauze and solution directly. Hydrated pollen grains were incubated in 100 mL germination medium (20 mM MES, 3 mM Ca(NO_3_)_2_, 1 mM KCl, 0.8 mM MgSO_4_, 1.6 mM boric acid, 24% PEG 4000, 2.5% sucrose, pH 6.0; osmotic pressure, 1253.33 ± 2.33 mOsmol/kg H_2_O) in a Petri dish (150 × 25 mm) at 25°C in the dark with shaking at 90 rpm (Tang et al., 2002; Zhao et al., 2013). During germination, 1 mL medium was took out at regular intervals, centrifuged to collect germinating pollen grains, then transferred to 1 mL Carnoy’s fluid (three parts of absolute ethyl alcohol, one part of acetic acid) for treatment of 30 min. Thereafter, these treated pollen grains or tubes were stained with 4′,6-diamino-2-phenylindole (DAPI; Molecular Probes) and observed under a microscope (Zeiss Axio Imager A1).

### Isolation of GCs

An improved two-step osmotic shock was used to release GCs from JGPGs and all procedures were performed at room temperature (Zhao et al., 2013). Two aliquots of pollen grains 90 mg each were germinated in 100 mL germination medium as described above for ∼20 min until the pollen tubes emerged but were shorter than the diameter of the grains. JGPGs were harvested through a Büchner funnel (100 mm in diameter) with 11-μm hydrated nylon mesh (Millipore, NY1100010) with the help of an aspirator pump, then rinsed with osmotic shock solution (15.3% sucrose, 1% bovine serum albumin [BSA], 531.33 ± 3.84 mOsmol/kg H_2_O) to clean germination medium, which would affect the result of osmotic shock. The collected JGPGs were immediately transferred to 80 mL fresh osmotic shock solution and incubated for 10 min to burst tubes and release GCs. Cell debris was removed by sieving the mixture through a hydrated 11-μm nylon mesh. The filtrate containing GCs was equally divided into two centrifugation tubes, and centrifuged at 850 *g* for 4 min to collect GCs. To avoid loss of GCs, we retained 10 ml of the supernatant in each tube after centrifugation, then added 10 mL of isolation buffer 1 (IB1; 20 mM MES-KOH, 20 mM NaCl, 10 mM EDTANa_2_, 1 mM spermidine, 0.3 mM spermine, 2 mM DTT, 18% sucrose and 1% BSA, pH6.0) to suspend GCs. The suspension was supplemented with stock solution of cellulase “Onozuka” R-10 (Yakult) and macerozyme R-10 (Yakult; 0.4% each in IB2; IB2; 10 mM MES-KOH, 10 mM NaCl, 5 mM EDTANa_2_, 0.5 mM spermidine, 0.15 mM spermine, 1 mM DTT, 18% sucrose and 1% BSA, pH6.0) to a final concentration of 0.04% each enzyme, mixed gently and incubated for 15 min without shaking, and centrifuged at 850 *g* for 3 min to collect GCs, followed by a washing with IB2. Collected GCs were further purified on 23/32% Percoll density gradient (2 mL 23% Percoll and 3 mL 32% Percoll in IB2) by horizontal centrifugation at 1000 *g* for 40 min. After centrifugation, GCs partitioned at the interface of 23% and 32% Percoll were collected with use of a glass pipette and washed twice with 3 × volume IB2 followed by centrifugation at 950 *g* for 3 min each. The viability of isolated GCs was examined by fluorescein diacetate (FDA) staining. The purified GCs were snap-frozen in liquid nitrogen and stored at −80°C.

### Isolation of SCs

Sperm cells were isolated under room temperature as described by Xu et al. (2002) with modifications. In brief, three aliquots of pollen grains 60 mg each were cultured in 100 mL germination medium as described above for 10 h. After germination, medium was removed by use of hydrated 100-μm nylon mesh (Millipore, NY1H00010), and pollen tubes were washed with osmotic shock solution as for isolation of GCs, immediately transferred to low-osmotic enzyme solution (0.4% cellulase “Onozuka” R-10 and 0.2% macerozyme R-10 in osmotic shock solution), and incubated for 5 min to release SCs. Cell debris and ungerminated pollen grains were removed by use of hydrated 11-μm nylon mesh, and SCs in the filtrate were collected and washed as described in isolation of GCs. Thereafter, collected SCs were purified on 5 mL 23% Percoll gradient in IB2 by horizontal centrifugation at 1000 *g* for 30 min. SCs were enriched to the upper surface of 23% Percoll gradient and harvested by use of a glass pipette and washed twice with 3 × volume IB2 followed by centrifugation at 950 *g* for 3 min each. The viability of isolated SCs was examined by FDA staining. The purified SCs were snap-frozen in liquid nitrogen and stored at −80°C.

### Isolation of VN

All operations were performed on ice or at 4°C unless otherwise specified, and all solutions were pre-cooled on ice or at 4°C. Three aliquots of pollen grains 90 mg each were cultured in 100 mL germination medium as described above for 1.5 h. Pollen tubes were collected with a 20-μm hydrated nylon mesh (Millipore, NY2009000) at room temperature, rinsed with wash buffer (10 mM MOPS-NaOH, 2.5% sucrose, 9.5% mannitol, 5 mM EDTANa_2_, 1% BSA, pH7.2), and treated with 12 mL enzyme solution (0.5% cellulase “Onozuka” R-10, and 0.3% macerozyme R-10 in wash buffer) for 5 min to release VN. After removal of ungerminated pollen grains and cell debris with use of 20-μm hydrated nylon mesh, the filtrate containing VN was divided into four equal parts, loaded onto the surface of 3 mL 10% Percoll gradient in wash buffer each, then centrifuged at 1500 *g* for 30 min. VN in the upper surface of the gradient were collected by use of a glass pipette, snap-frozen in liquid nitrogen, then stored at −80°C.

## Results

### Dynamics of GCs and SCs During Culture *In Vitro*

**FIGURE 1:**
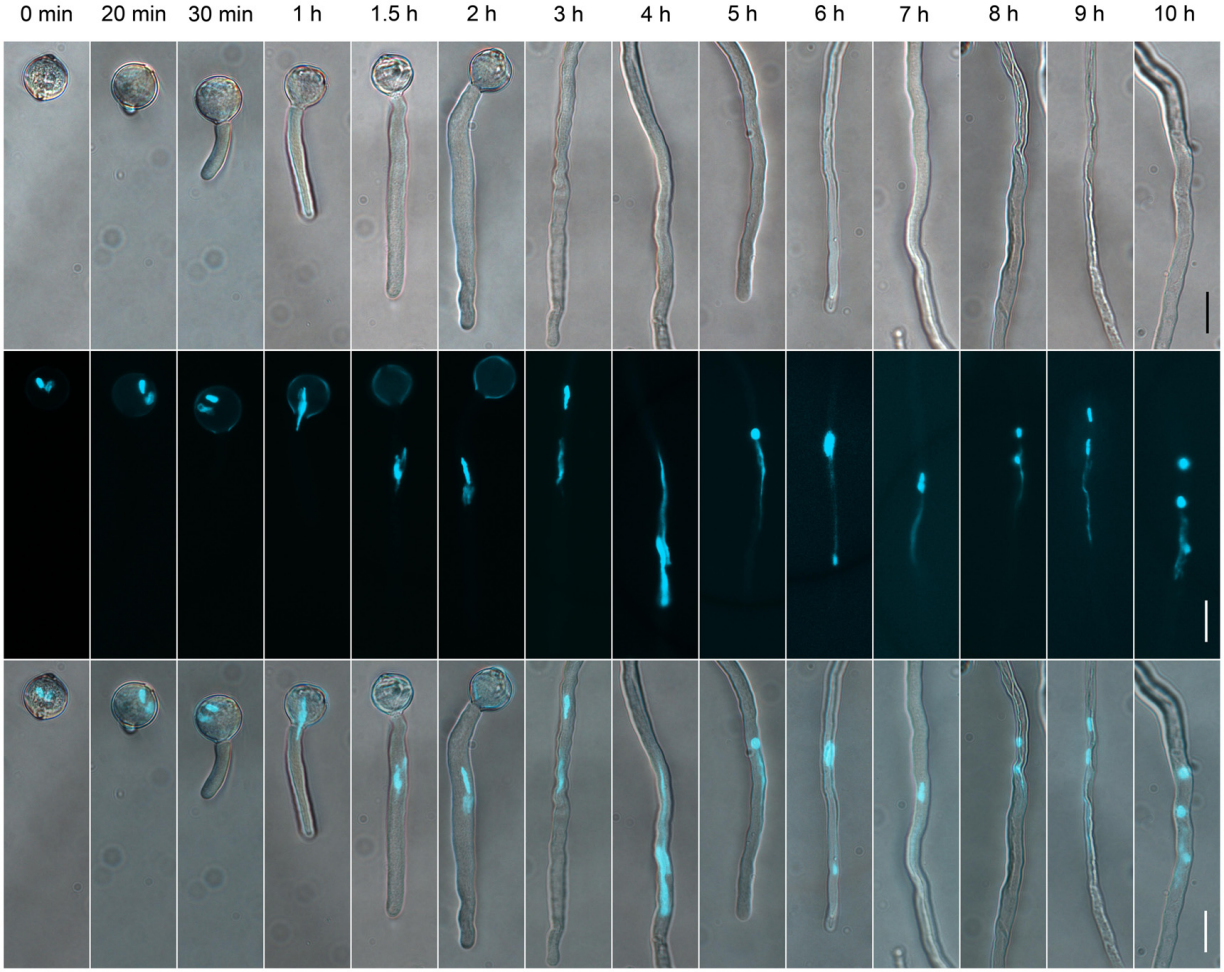
**The dynamics of generative cells (GCs) and sperm cells (SCs) during pollen germination and tube growth**. Pollen cultured at different times observed by differential interference contrast (DIC) microscopy (upper panel), or after 4′,6-diamino-2-phenylindole (DAPI) staining (middle panel). Merged images in the bottom panel. Scale bar: 20 μm.

Our experiments showed that low-temperature (−20°C) stored tomato pollen grains without prehydration germinated *in vitro* at low germination rate (Supplementary Table S1). To guarantee a high proportion of synchronously germinated tomato pollen grains after low-temperature storage and long-term growth of pollen tubes to allow generation of SCs in the tube *in vitro*, we optimized the pre-hydration condition of the stored pollen grains and culture condition of pollen tubes. We prehydrated low-temperature-stored tomato pollen grains using the saturated solution of Na_2_HPO_4_ to rescue the germination activity. Prehydration with the saturated solution for 4–8 h significantly increased germination rate of the pollen grains (Supplementary Table S1). Our culture conditions allowed for *in vitro* growth of pollen tubes for >10 h (**Figure 1**).

**FIGURE 2:**
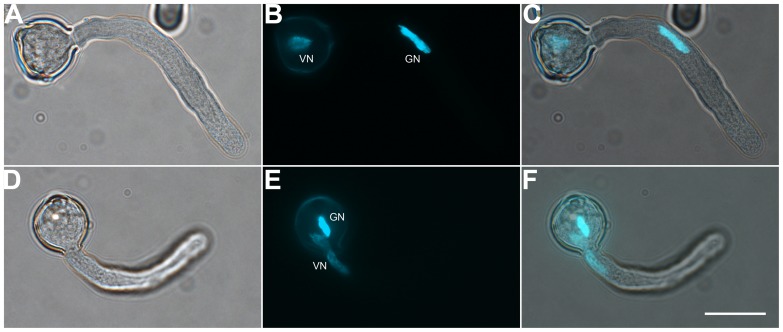
**Both vegetative cell nuclei (VN) and GCs can enter the pollen tube first**. Pollen germinated for 1 h observed by DIC microscopy **(A,D)** or after DAPI staining **(B,E)**. Images were merged **(C,F)**. DAPI-stained VN and GC nucleus (GN) in **(B)** and **(E)**. Scale bar: 20 μm.

To determine the suitable time of pollen tube growth for isolating GCs, SCs, and VN, we examined their dynamics during pollen germination and tube growth by DAPI staining. A bulge appeared at the germination aperture of hydrated pollen grains on culture for 20 min, and the bulge emerged as a morphologically visible pollen tube with a length shorter than or equal to the diameter of the pollen grain during 30-min culture (**Figure 1**). With increased culture time, GCs and VN began to move into the tube at 1 h and completely entered the tube at 1.5 h. We used DAPI staining to determine the movement order of VN and GCs (**Figure 2**). Among 126 surveyed pollen grains, for 68, VN entered the tube first, and for 58, GCs entered first. Therefore, during tomato pollen tube growth, VN and GCs may move into the tube in a random order. Furthermore, for GCs, 77.8% completed mitosis to generate SCs at 8 h, 84.2% at 9 h, and 92.4% at 10 h (Supplementary Table S2).

### Release and Purification of GCs

**FIGURE 3:**
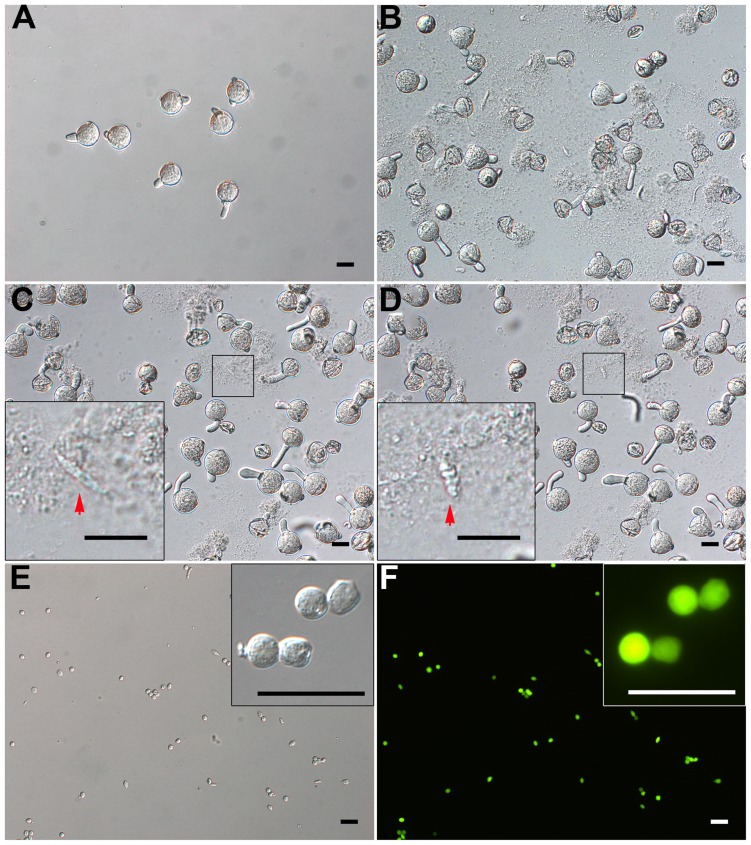
**Isolation of GCs from just-germinated pollen grains. (A)** Pollen grains were geminated *in vitro* for 20 min, and most produced pollen tubes. **(B)** 10-min osmotic shock of germinated pollen grains in osmotic shock solution led to a burst of most of pollen tubes. The inset image in **(C)** and **(D)** shows change of just-released GC from spindle-shaped **(C)** to oval-shaped **(D)**. **(E,F)** DIC microscopy **(E)** and viability with fluorescein diacetate (FDA) staining **(F)** of purified GCs. Close-up of GCs in inset images **(E,F)**. Scale bar: 20 μm.

Tomato pollen grains could not burst directly with osmotic shock and also could not germinate in a sucrose solution alone (data not shown). So, we developed a modified two-step method. We incubated prehydrated tomato pollen grains in germination medium for 20 min, when a bulge appeared at the aperture (**Figures 1** and **3A**), then osmotically shocked the JGPGs, which were sensitive to the low-osmotic shock (**Figure 3B**).

When JGPGs were transferred into low-osmotic solution, the tube burst, and GCs, along with the cytoplasm, were emitted (**Figures 3C,D**). The just-released GCs underwent a change from spindle- to oval-shaped (**Figures 3C,D**). This change may be associated with the microtubule cytoskeleton, which was dynamic in response to environmental conditions and is important to determine the shape of GCs (Zhou et al., 1990). When incubated in the low-osmotic solution for 10 min, 62.9% (680/1082) of pollen tubes burst (**Figure 3B**). The released GCs were intact in the low-osmotic solution up to 1 h, but most GCs appeared to break after 1 h (data not shown). To maintain GC integrity, we added IB1 into the filtrate containing GCs to neutralize the low osmotic shock in a short time (<1 h). The suspension was used for purifying GCs.

Furthermore, we treated the suspension with cellulase and macerozyme at a low concentration, which had no effect on viability of GCs but increased the efficiency in removing cell debris at subsequent gradient centrifugation. GCs were enriched at the interface of 23% and 32% Percoll gradient, with cell debris on the upper interface of 23% Percoll. The isolated GCs were viable on FDA staining (**Figures 3E,F**) and had no VN contamination, as confirmed by propidium iodide (PI) staining (viable GCs cannot be stained by PI; Supplementary Figures S1A,B). Finally, we obtained about 1.5 million GCs from 180 mg of initiated mature pollen grains (about 18 million grains, in that 0.1 million tomato pollen grains is about 1 mg).

### Release and Purification of SCs

**FIGURE 4:**
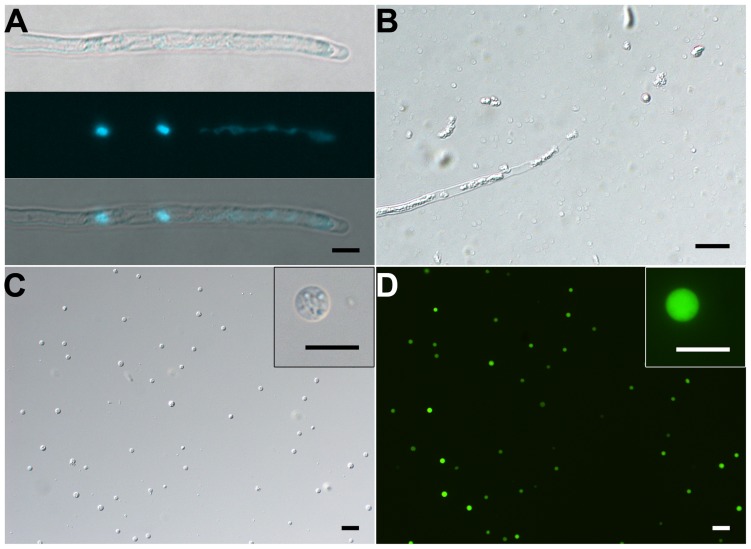
**Isolation of SCs from 10-h–cultured pollen tubes. (A)** DIC (upper) and DAPI staining (middle) and merged (bottom) images of pollen tube. **(B)** Representative enzymolysis-treated pollen tubes. **(C,D)** DIC microscopy **(C)** and viability of purified SCs with FDA staining **(D)**. The inset images in **(C)** and **(D)** are close-ups of the SC. Scale bar: 20 μm in **(A)** to **(D)**, 10 μm in inset images of **(C)** and **(D)**.

Successful isolation of SCs from *in vitro*-cultured pollen tubes depends on the formation of SCs in the growing tubes. We isolated SCs from 10-h-cultured pollen tubes, in which GCs had completed mitosis to generate SCs (see above, **Figure 4A**; Supplementary Table S2). To decrease the possible contamination of GCs, we collected long pollen tubes using a large pore-size nylon mesh (100 μm), which allowed ungerminated pollen grains and shorter pollen tubes to pass through. We found that osmotic shock alone did not burst the long tubes efficiently (data not shown), and a modified low osmotic solution with cellulase and macerozyme was efficient to burst the tube (**Figure 4B**). After removal of cell debris, SCs in filtrate could be enriched with a layer of 23% Percoll. Finally, we obtained about 2 million viable SCs at high purity from 180 mg initiated pollen grains (**Figures 4C,D**), with no VN contamination (Supplementary Figures S1C,D).

### Release and Purification of VN

**FIGURE 5:**
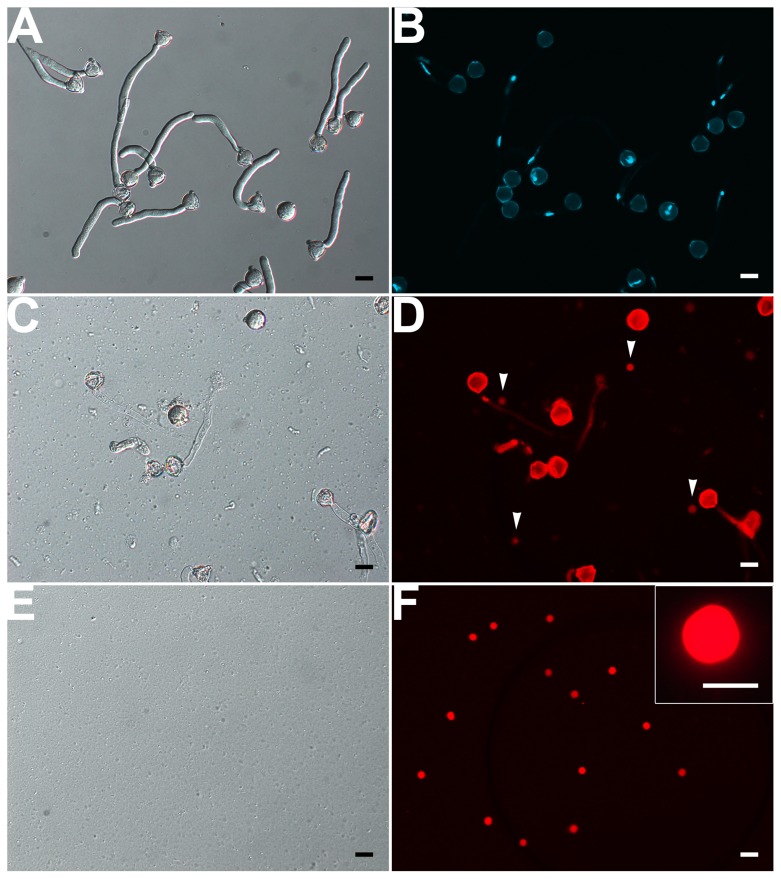
**Isolation of VN from pollen tubes. (A,B)** DIC **(A)** and DAPI staining **(B)** of pollen tubes cultured *in vitro* for 1.5 h. **(C,D)** DIC microscopy **(C)** and propodium iodide (PI) staining **(D)** of enzymolysis-treated pollen tubes; arrows indicate released VNs **(D)**. **(E,F)** DIC microscopy **(E)** and PI staining **(F)** of purified VN. Inset image in **(F)** is close-up of a PI-stained VN. Scale bar: 20 μm in **(A)** to **(F)**, 10 μm in the inset image **(F)**.

Our results showed that VN was fragile and disrupted quickly as released to medium at room temperature. Repeated pipetting also led to its complete disruption (Supplementary Figures S2A,B,C). Therefore, no VN contamination was present in isolated GCs and SCs (see above). We solved the bottleneck of VN isolation by (1) keeping all operations at 4°C or on ice, (2) avoiding pipetting as much as possible, and (3) using 1.5-h-cultured pollen tubes in which VN had moved into the tube (**Figures 1** and **5A,B**), for easier release of VN (**Figures 5C,D**). Furthermore, additional washing as well as passing through Percoll on gradient centrifugation disrupted VN (Supplementary Figures S2A,D), so we used only a hydrated nylon net filter to remove pollen grains and cell debris and then enriched VN by using a layer of 10% Percoll. These measures allowed for isolation of VN without GC contamination (**Figures 5E,F**). Using this protocol, we obtained 10 million VN from 270 mg initiated pollen grains.

## Discussion

**FIGURE 6:**
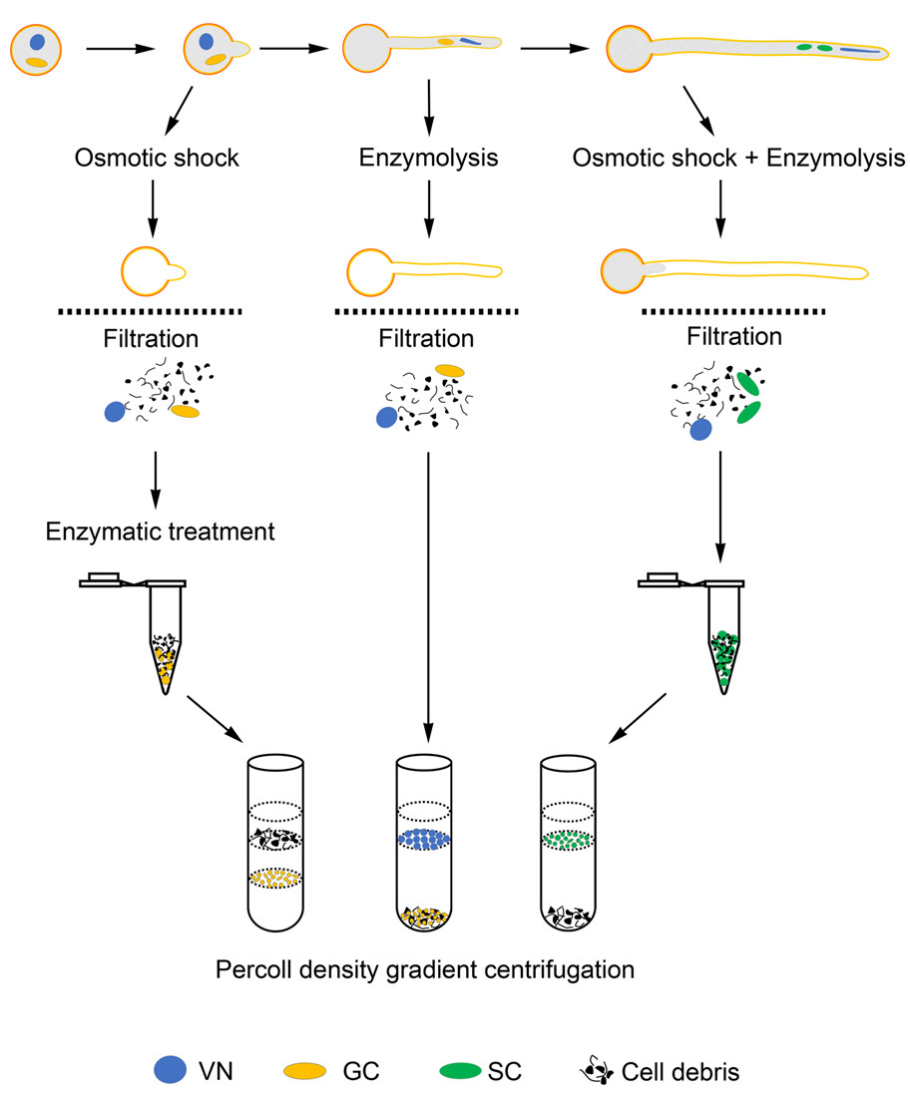
**Schematic of methods to isolate GCs, SCs, and VN**.

We have optimized the conditions allowing for growth of pollen tubes for more than 10 h and generation of SCs in tubes, as well as conditions affecting rupture of pollen grains (tubes) and release of cytoplasm, GCs, SCs and VN into medium. Finally, we developed methods to isolate GCs, SCs, and VN from JGPGs and 1.5-h– and 10-h–cultured pollen tubes, respectively (**Figure 6**). These methods allowed for isolating large amounts of GCs, SCs, and VN at high purity.

### Culture Conditions for Low-Temperature–Stored Pollen Grains and Long-Term–Cultured Pollen Tubes

Previous study established the condition for *in vitro* germination of fresh pollen grains from tomato (Tang et al., 2002), but under the condition, low-temperature–stored tomato pollen grains did not germinate well (Supplementary Table S1). Generally, pre-hydration is required for rescuing the viability of low-temperature-stored pollen grains, such as from *Rosa*, *Pistacia vera* L., *Gladiolus* sp. and *Brassica rapa* (Visser et al., 1977; Golan-Goldhirsh et al., 1991; Rajasekharan et al., 1994; Sato et al., 1998). The prehydration was usually actualized with water or saturated salt solution, which generated a fixed relative humidity in a chamber at a certain temperature. We found that a saturated solution of Na_2_HPO_4_ was suitable for prehydrating tomato pollen grains. The saturated solution was previously used to prehydrate *B. rapa* pollen (Sato et al., 1998), and could result in 95% relative humidity in a chamber at 25°C, and tomato pollen grains rescued under this condition germinated synchronously (**Figure 3A**), which was important for synchronous pollen tube growth and GC division. Pollen density also was an important factor affecting germination in tomato, and increased density led to increased germination percentage (Supplementary Figure S3), which agrees with observations in other species such as *Arabidopsis* (Boavida and McCormick, 2007), *Nicotiana, B. oleracea*, and *Betula pendula* (Roberts et al., 1983; Jahnen et al., 1989; Pasonen and Käpylä, 1998; Chen et al., 2000). Furthermore, we evaluated the effect of loaded pollen grain amount in a given volume germination medium on the integrity of pollen tubes during long-term culture (>3 h) by examining cell debris in the medium (Supplementary Figure S4). Cell debris was barely observed with ≤4 mg pollen grains used (Supplementary Figures S4A–D), and substantial with 5 mg pollen grains (Supplementary Figure S4E). Thus, the amount of pollen grains in a given volume germination medium is crucial to the integrity of long-term–cultured pollen tubes, and 3 mg pollen grains loaded in 5 mL germination medium was appropriate for long-term culture of pollen tubes.

### Methods to Release GCs, SCs, and VN

Four major methods were used previously to break pollen grains or tubes: mechanical grinding, one-step and two-step osmotic shock and enzyme digestion (Russell, 1986, 1991; Zhou, 1988; Russell et al., 1990; Theunis et al., 1991; Chaboud and Perez, 1992; Xu et al., 2002; Borges et al., 2008; Zhao et al., 2013). The mechanical grinding had relatively low efficiency for breaking tomato pollen grains and produced a large amount of debris, which interfered with further purification. One-step or two-step osmotic shock is usually used to break pollen grains (tubes) and release target cells. The former breaks pollen grains and releases target cells simultaneously using a low-osmotic solution (Russell, 1986). The latter first makes the grain germinate in a sucrose solution, then release target cells under low-osmotic shock with a diluted sucrose solution (Zhou, 1988; Wu and Zhou, 1991). Tomato pollen grains were insensitive to osmotic shock and even did not burst under osmotic shock of water, which is similar to pollen grains from *Vicia faba*, *B. napus*, and *L. davidii* var. *unicdor* (Zhou, 1988; Taylor et al., 1991; Zhao et al., 2013), thus suggesting a complicated mechanism of pollen grain burst for different species and individual methods needed for different species.

We choose the two-step osmotic shock to release GCs. Components of osmotic shock solution affected the appearance of released cytoplasm, which affected the following purification of GCs. The released cytoplasm appeared not to conglutinate when the solution had only 15.3% sucrose (Supplementary Figure S5A) but appeared to conglutinate with shock solution containing MES or MOPS regardless of concentration (Supplementary Figures S5B–I). Acidic pH could aggravate this situation (Supplementary Figures S5D,J,L). The phenomenon of cytoplasm clumping was also observed under high concentration of CaCl_2_ in a previous study of isolating SCs from pollen tubes of *N. tabacum* (Tian and Russell, 1997). We chose sucrose and BSA as the components of the osmotic shock solution. The use of BSA in all solutions except the germination medium aimed to protect GCs against damage because we found that GCs could remain intact in the shock solution with BSA up to 1 h but for only a few minutes in a shock solution without BSA; GCs from several species such as *V. faba* could remain intact in simple sucrose shock solution until being purified (Zhou, 1988).

However, pollen tubes cultured *in vitro* for 10 h, which we used to isolate SCs, were not as sensitive as pollen tubes cultured for a short time to osmotic shock described above. So we developed a modified shock solution containing cellulase and macroenzyme. The two enzymes are usually applied to digest hemicelluloses, cellulose and pectin, the main components of the pollen tube wall (Taylor and Hepler, 1997; Cheung and Wu, 2008; Rounds and Bezanilla, 2013). This modified shock solution broke the long pollen tubes and released SCs efficiently (**Figure 4B**). Why pollen tubes had different sensitivity to osmotic shock with long- and short-term culture needs further studies.

In contrast to reports of GCs and SCs, we have limited reports of VN isolation (Wever and Takats, 1971; LaFountain and Mascarenhas, 1972; Ueda and Tanaka, 1994; Borges et al., 2012; Calarco et al., 2012). These reports did not describe the stability of isolated VN or the effect of environmental conditions on the stability. We found that VN was fragile *in vitro* (Supplementary Figure S2) and easily ruptured at room temperature but was relatively stable at 4°C. However, at low temperature, pollen tubes were not sensitive to osmotic shock. In this situation, enzyme digestion was found efficient to break pollen tubes and release VN but depended on a suitable buffer. We found that pH value was a crucial factor of the buffer. The pH value affected the appearance of the released cytoplasm: conglutination appeared at acidic pH and not at alkalinous pH (Supplementary Figure S6).

### Measures to Guarantee the Purity of GCs, SCs, and VN

Previous reports mainly described isolation of SCs from tricellular pollen or GCs from bicellular pollen (Russell, 1986; Dupuis et al., 1987; Zhou, 1988; Southworth and Knox, 1989; Yang and Zhou, 1989; Xu et al., 2002; Engel et al., 2003), or along with VN (Borges et al., 2012). For these cases, possible contaminants were VN for isolated SCs or GCs and vice versa. Such purity evaluation was relatively simple. Most previous works evaluated the purity of isolated SCs or GCs based on their morphologic features observed on microscopy (Zhou, 1988; Southworth and Knox, 1989; Yang and Zhou, 1989; Xu et al., 2002; Engel et al., 2003). Only the study of *Arabidopsis* used SC- and VN-specific markers to estimate the purity of isolated SCs and VN because of available markers for this species (Borges et al., 2008, 2012).

An evaluation of the purity of isolated GCs, SCs, or VN from a species was relatively lacking in early studies. We found that a combination of PI staining and differential interference contrast (DIC) microscope observation was efficient to evaluate VN contamination of GCs or SCs and vice versa. VN was not observed on DIC microscopy but were detectable with PI staining. In contrast, viable GCs and SCs were easily observed on DIC microscopy but undetectable with PI staining. Using the combination, we did not find VN contamination in isolated GCs and SCs or GC and SC contamination in isolated VN (Supplementary Figure S1, **Figures 5E,F**). We considered several measures to eliminate this contamination in designing methods to isolate GCs, SCs, and VN. (1) GCs or SCs were stable and isolated at room temperature, but under this temperature, VN was fragile and broke quickly. (2) GCs, SCs and VN had different density on Percoll gradient and could be enriched with different gradient ingredients. These measures could eliminate VN contamination in isolated GCs and SCs and vice versus (Supplementary Figure S1, **Figures 5E,F**).

A major challenge in methodology was how to get SCs at high purity. Here, besides the use of high synchronous pollen tubes, of which 92.4% generated SCs (Supplementary Table S2), and the measures above, we collected long pollen tubes by using a large-pore mesh (100 μm in pore diameter), which excluded ungerminated pollen grains (diameter of about 20 μm) and short pollen tubes. Thus, the methods could obtain SCs at high purity. However, tools to distinguish GCs and SCs are lacking because of their similar morphology under light microscope in tomato and other most species and lack of molecular markers.

## Conclusion

Saturated Na_2_HPO_4_ solution was suitable for pre-hydration of low-temperature–stored tomato pollen grains, and the prehydrated pollen grains germinated synchronously. The loaded amount of 0.6 mg pollen grains per mL allowed pollen tubes to grow for more than 10 h, and more than 92% GCs completed mitosis to generate SCs. GCs or SCs were stable and could be isolated at room temperature, whereas under the same temperature, VN was fragile and broke quickly *in vitro*. GCs, SCs, and VN had different density on Percoll gradient, and could be enriched with different gradient ingredients. Thus, we have established methods to isolate GCs and VN from just-germinated pollen grains and 1.5-h–cultured pollen tubes, respectively, and SCs from 10-h–cultured pollen tubes. Using these methods, we could obtain 1.5 million GCs and 2 million SCs each from 180 mg initiated pollen grains, and 10 million VN from 270 mg initiated pollen grains, for higher productivity as compared with previous reports of other species.

## Conflict of Interest Statement

The authors declare that the research was conducted in the absence of any commercial or financial relationships that could be construed as a potential conflict of interest.

## References

[B1] Berger F. Twell D. (2011). Germline specification and function in plants. *Annu. Rev. Plant Biol.* 62 461–484. 10.1146/annurev-arplant-042110-103824 21332359

[B2] Boavida L. C. McCormick S. (2007). Temperature as a determinant factor for increased and reproducible *in vitro* pollen germination in *Arabidopsis thaliana*. *Plant J.* 52 570–582. 10.1111/j.1365-313X.2007.03248.x 17764500

[B3] Borg M. Brownfield L. Khatab H. Sidorova A. Lingaya M. Twell D. (2011). The R2R3 MYB transcription factor DUO1 activates a male germline-specific regulon essential for sperm cell differentiation in *Arabidopsis*. *Plant Cell* 23 534–549. 10.1105/tpc.110.081059 21285328 PMC3077786

[B4] Borg M. Brownfield L. Twell D. (2009). Male gametophyte development: a molecular perspective. *J. Exp. Bot.* 60 1465–1478. 10.1093/Jxb/Ern355 19213812

[B5] Borg M. Rutley N. Kagale S. Hamamura Y. Gherghinoiu M. Kumar S. (2014). An EAR-dependent regulatory module promotes male germ cell division and sperm fertility in *Arabidopsis*. *Plant Cell* 26 2098–2113. 10.1105/tpc.114.124743 24876252 PMC4079371

[B6] Borges F. Gardner R. Lopes T. Calarco J. P. Boavida L. C. Slotkin R. K. (2012). FACS-based purification of *Arabidopsis* microspores, sperm cells and vegetative nuclei. *Plant Methods* 8:44 10.1186/1746-4811-8-44 PMC3502443 23075219

[B7] Borges F. Gomes G. Gardner R. Moreno N. McCormick S. Feijó J. A. (2008). Comparative transcriptomics of *Arabidopsis* sperm cells. *Plant Physiol.* 148 1168–1181. 10.1104/pp.108.125229 18667720 PMC2556834

[B8] Brownfield L. Hafidh S. Borg M. Sidorova A. Mori T. Twell D. (2009a). A plant germline-specific integrator of sperm specification and cell cycle progression. *PLoS Genet.* 5:e1000430 10.1371/journal.pgen.1000430 PMC2653642 19300502

[B9] Brownfield L. Hafidh S. Durbarry A. Khatab H. Sidorova A. Doerner P. (2009b). *Arabidopsis* DUO POLLEN3 is a key regulator of male germline development and embryogenesis. *Plant Cell* 21 1940–1956. 10.1105/tpc.109.066373 19638475 PMC2729611

[B10] Calarco J. P. Borges F. Donoghue M. T. Van Ex F. Jullien P. E. Lopes T. (2012). Reprogramming of DNA methylation in pollen guides epigenetic inheritance via small RNA. *Cell* 151 194–205. 10.1016/j.cell.2012.09.001 23000270 PMC3697483

[B11] Chaboud A. Perez R. (1992). Generative cells and male gametes: isolation, physiology, and biochemistry. *Int. Rev. Cytol.* 140 205–232. 10.1016/S0074-7696(08)61098-0

[B12] Chen Y. F. Matsubayashi Y. Sakagami Y. (2000). Peptide growth factor phytosulfokine-α contributes to the pollen population effect. *Planta* 211 752–755. 10.1007/s004250000370 11089690

[B13] Cheung A. Y. Wu H. M. (2008). Structural and signaling networks for the polar cell growth machinery in pollen tubes. *Annu. Rev. Plant Biol.* 59 547–572. 10.1146/annurev.arplant.59.032607.092921 18444907

[B14] Dai S. J. Chen T. T. Chong K. Xue Y. B. Liu S. Q. Wang T. (2007). Proteomics identification of differentially expressed proteins associated with pollen germination and tube growth reveals characteristics of germinated *Oryza sativa* pollen. *Mol. Cell. Proteomics* 6 207–230. 10.1074/mcp.M600146-MCP200 17132620

[B15] Dai S. Li L. Chen T. Chong K. Xue Y. Wang T. (2006). Proteomic analyses of *Oryza sativa* mature pollen reveal novel proteins associated with pollen germination and tube growth. *Proteomics* 6 2504–2529. 10.1002/pmic.200401351 16548068

[B16] Dupuis I. Roeckel P. Matthys-Rochon E. Dumas C. (1987). Procedure to isolate viable sperm cells from corn (*Zea mays* L.) pollen grains. *Plant Physiol.* 85 876–878. 10.1104/pp.85.4.876 16665823 PMC1054361

[B17] Engel M. L. Chaboud A. Dumas C. McCormick S. (2003). Sperm cells of *Zea mays* have a complex complement of mRNAs. *Plant J.* 34 697–707. 10.1046/j.1365-313X.2003.01761.x 12787250

[B18] Filichkin S. A. Leonard J. M. Monteros A. Liu P. P. Nonogaki H. (2004). A novel endo-*β*-mannanase gene in tomato LeMAN5 is associated with anther and pollen development. *Plant Physiol.* 134 1080–1087. 10.1104/pp.103.035998 14976239 PMC389932

[B19] Golan-Goldhirsh A. Schmidhalter U. Müller M. Oertli J. J. (1991). Germination of *Pistacia vera* L. pollen in liquid medium. *Sex. Plant Reprod.* 4 182–187. 10.1007/BF00190002

[B20] Grant-Downton R. Kourmpetli S. Hafidh S. Khatab H. Le Trionnaire G. Dickinson H. (2013). Artificial microRNAs reveal cell-specific differences in small RNA activity in pollen. *Curr. Biol.* 23 R599–R601. 10.1016/j.cub.2013.05.055 23885870

[B21] Holmes-Davis R. Tanaka C. K. Vensel W. H. Hurkman W. J. McCormick S. (2005). Proteome mapping of mature pollen of *Arabidopsis thaliana*. *Proteomics* 5 4864–4884. 10.1002/pmic.200402011 16247729

[B22] Honys D. Twell D. (2004). Transcriptome analysis of haploid male gametophyte development in *Arabidopsis*. *Genome Biol.* 5 R85 10.1186/Gb-2004-5-11-R85 PMC545776 15535861

[B23] Jahnen W. Lush W. M. Clarke A. E. (1989). Inhibition of *in vitro* pollen tube growth by isolated S-glycoproteins of *Nicotiana alata*. *Plant Cell* 1 501–510. 10.1105/tpc.1.5.501 12359898 PMC159783

[B24] LaFountain K. L. Mascarenhas J. P. (1972). Isolation of vegetative and generative nuclei from pollen tubes. *Exp. Cell Res.* 73 233–236. 10.1016/0014-4827(72)90125-5 5036992

[B25] McCormick S. (1993). Male gametophyte development. *Plant Cell* 5 1265–1275. 10.2307/3869779 12271026 PMC160359

[B26] Muschietti J. Dircks L. Vancanneyt G. McCormick S. (1994). Lat52 protein is essential for tomato pollen development: pollen expressing antisense *Lat52* RNA hydrates and germinates abnormally and cannot achieve fertilization. *Plant J.* 6 321–338. 10.1046/j.1365-313X.1994.06030321.x 7920720

[B27] Obermeyer G. Fragner L. Lang V. Weckwerth W. (2013). Dynamic adaption of metabolic pathways during germination and growth of lily pollen tubes after inhibition of the electron transport chain. *Plant Physiol.* 162 1822–1833. 10.1104/pp.113.219857 23660836 PMC3729764

[B28] Pasonen H. L. Käpylä M. (1998). Pollen-pollen interactions in *Betula pendula in vitro*. *New Phytol.* 138 481–487. 10.1046/j.1469-8137.1998.00135.x

[B29] Rajasekharan P. E. Rao T. M. Janakiram T. Ganeshan S. (1994). Freeze preservation of gladiolus pollen. *Euphytica* 80 105–109. 10.1007/Bf00039304

[B30] Roberts I. N. Gaude T. C. Harrod G. Dickinson H. G. (1983). Pollen-stigma interactions in *Brassica oleracea*; a new pollen germination medium and its use in elucidating the mechanism of self incompatibility. *Theor. Appl. Genet.* 65 231–238. 10.1007/Bf00308074 24263420

[B31] Rounds C. M. Bezanilla M. (2013). Growth mechanisms in tip-growing plant cells. *Annu. Rev. Plant Biol.* 64 243–265. 10.1146/annurev-arplant-050312-120150 23451782

[B32] Russell S. D. (1986). Isolation of sperm cells from the pollen of *Plumbago zeylanica*. *Plant Physiol.* 81 317–319. 10.1104/pp.81.1.317 16664799 PMC1075328

[B33] Russell S. D. (1991). Isolation and characterization of sperm cells in flowering plants. *Annu. Rev. Plant Physiol. Plant Mol. Biol.* 42 189–204. 10.1146/annurev.arplant.42.1.189

[B34] Russell S. D. Cresti M. Dumas C. (1990). Recent progress on sperm characterization in flowering plants. *Physiol. Plant.* 80 669–676. 10.1034/j.1399-3054.1990.800427.x

[B35] Russell S. D. Gou X. P. Wong C. E. Wang X. K. Yuan T. Wei X. P. (2012). Genomic profiling of rice sperm cell transcripts reveals conserved and distinct elements in the flowering plant male germ lineage. *New Phytol.* 195 560–573. 10.1111/j.1469-8137.2012.04199.x 22716952

[B36] Rutley N. Twell D. (2015). A decade of pollen transcriptomics. *Plant Reprod.* 28 73–89. 10.1007/s00497-015-0261-7 25761645 PMC4432081

[B37] Sato S. Katoh N. Iwai S. Hagimori M. (1998). Establishment of reliable methods of *in vitro* pollen germination and pollen preservation of *Brassica rapa* (syn. B campestris). *Euphytica* 103 29–33. 10.1023/A:1018381417657

[B38] Sato S. Tabata S. Hirakawa H. Asamizu E. Shirasawa K. Isobe S. (2012). The tomato genome sequence provides insights into fleshy fruit evolution. *Nature* 485 635–641. 10.1038/Nature11119 22660326 PMC3378239

[B39] Slotkin R. K. Vaughn M. Borges F. Tanurdžić M. Becker J. D. Feijó J. A. (2009). Epigenetic reprogramming and small RNA silencing of transposable elements in pollen. *Cell* 136 461–472. 10.1016/j.cell.2008.12.038 19203581 PMC2661848

[B40] Southworth D. Knox R. B. (1989). Flowering plant sperm cells: isolation from pollen of *Gerbera jamesonii* (Asteraceae). *Plant Sci.* 60 273–277. 10.1016/0168-9452(89)90177-5

[B41] Tang W. H. Ezcurra I. Muschietti J. McCormick S. (2002). A cysteine-rich extracellular protein, LAT52 interacts with the extracellular domain of the pollen receptor kinase LePRK2. *Plant Cell* 14 2277–2287. 10.1105/Tpc.003103 12215520 PMC150770

[B42] Taylor L. P. Hepler P. K. (1997). Pollen germination and tube growth. *Annu. Rev. Plant Physiol. Plant Mol. Biol.* 48 461–491. 10.1146/annurev.arplant.48.1.461 15012271

[B43] Taylor P. E. Kenrick J. Blomstedt C. K. Knox R. B. (1991). Sperm cells of the pollen tubes of *Brassica*: ultrastructure and isolation. *Sex. Plant Reprod.* 4 226–234. 10.1007/BF00190009

[B44] Theunis C. H. Pierson E. S. Cresti M. (1991). Isolation of male and female gametes in higher plants. *Sex. Plant Reprod.* 4 145–154. 10.1007/BF00189998

[B45] Tian H. Q. Russell S. D. (1997). Micromanipulation of male and female gametes of *Nicotiana tabacum* 1. Isolation of gametes. *Plant Cell Rep.* 16 555–560. 10.1007/BF01142323 30727578

[B46] Twell D. (2011). Male gametogenesis and germline specification in flowering plants. *Sex. Plant Reprod.* 24 149–160. 10.1007/s00497-010-0157-5 21103996

[B47] Twell D. Park S. K. Lalanne E. (1998). Asymmetric division and cell-fate determination in developing pollen. *Trends Plant Sci.* 3 305–310. 10.1016/S1360-1385(98)01277-1

[B48] Twell D. Yamaguchi J. McCormick S. (1990). Pollen-specific gene expression in transgenic plants: coordinate regulation of two different tomato gene promoters during microsporogenesis. *Development* 109 705–713. 2401221 10.1242/dev.109.3.705

[B49] Twell D. Yamaguchi J. Wing R. A. Ushiba J. McCormick S. (1991). Promoter analysis of genes that are coordinately expressed during pollen development reveals pollen-specific enhancer sequences and shared regulatory elements. *Genes Dev.* 5 496–507. 10.1101/Gad.5.3.496 1840556

[B50] Ueda K. Tanaka I. (1994). The basic proteins of male gametic nuclei isolated from pollen grains of *Lilium longiflorum*. *Planta* 192 446–452. 10.1007/BF00198582

[B51] Visser T. De Vries D. P. Welles G. W. H. Scheurink J. A. M. (1977). Hybrid tea-rose pollen. I. Germination and storage. *Euphytica* 26 721–728. 10.1007/Bf00021697

[B52] Wang Y. Zhang W. Z. Song L. F. Zou J. J. Su Z. Wu W. H. (2008). Transcriptome analyses show changes in gene expression to accompany pollen germination and tube growth in *Arabidopsis*. *Plant Physiol.* 148 1201–1211. 10.1104/pp.108.126375 18775970 PMC2577266

[B53] Wei L. Q. Xu W. Y. Deng Z. Y. Su Z. Xue Y. B. Wang T. (2010). Genome-scale analysis and comparison of gene expression profiles in developing and germinated pollen in *Oryza sativa*. *BMC Genomics* 11:338 10.1186/1471-2164-11-338 PMC2895629 20507633

[B54] Wei L. Q. Yan L. F. Wang T. (2011). Deep sequencing on genome-wide scale reveals the unique composition and expression patterns of microRNAs in developing pollen of *Oryza sativa*. *Genome Biol.* 12:R53 10.1186/Gb-2011-12-6-R53 PMC3218841 21679406

[B55] Wever G. H. Takats S. T. (1971). Isolation and separation of S-competent and S-incompetent nuclei from *Tradescantia* pollen grains. *Exp. Cell Res.* 69 29–32. 10.1016/0014-4827(71)90306-5 5124488

[B56] Wu X. Zhou C. (1991). A comparative study on methods for isolation of generative cell in various angiosperm species. *Acta Biol. Exp. Sin.* 24 15–23.

[B57] Xu H. P. Weterings K. Vriezen W. Feron R. Xue Y. B. Derksen J. (2002). Isolation and characterization of male-germ-cell transcripts in *Nicotiana tabacum*. *Sex. Plant Reprod.* 14 339–346. 10.1007/s00497-002-0128-6

[B58] Yang H. Y. Zhou C. (1989). Isolation of viable sperms from pollen of *Brassics napus, Zea mays* and *Secale cereale*. *Chin. J. Bot.* 1 80–84.

[B59] Zhao X. Yang N. Wang T. (2013). Comparative proteomic analysis of generative and sperm cells reveals molecular characteristics associated with sperm development and function specialization. *J. Proteome Res.* 12 5058–5071. 10.1021/pr400291p 23879389

[B60] Zhou C. (1988). Isolation and purification of generative cells from fresh pollen of *Vicia faba* L. *Plant Cell Rep.* 7 107–110. 10.1007/BF00270116 24241544

[B61] Zhou C. Zee S.Y. Yang H. Y. (1990). Microtubule organization of in situ and isolated generative cells in *Zephyranthes granditlora* Lindl. *Sex. Plant Reprod.* 3 213–218. 10.1007/BF00202877

