# Supplementary material for: Methods to isolate a large amount of generative cells, sperm cells and vegetative nuclei from tomato pollen for “omics” analysis
Source: Front Plant Sci. 2015 Jun 2;6:391. doi: 10.3389/fpls.2015.00391 (PMC4451641; doi:10.3389/fpls.2015.00391)
Supplement: Supplementary file 3 [file Image_1.PDF]

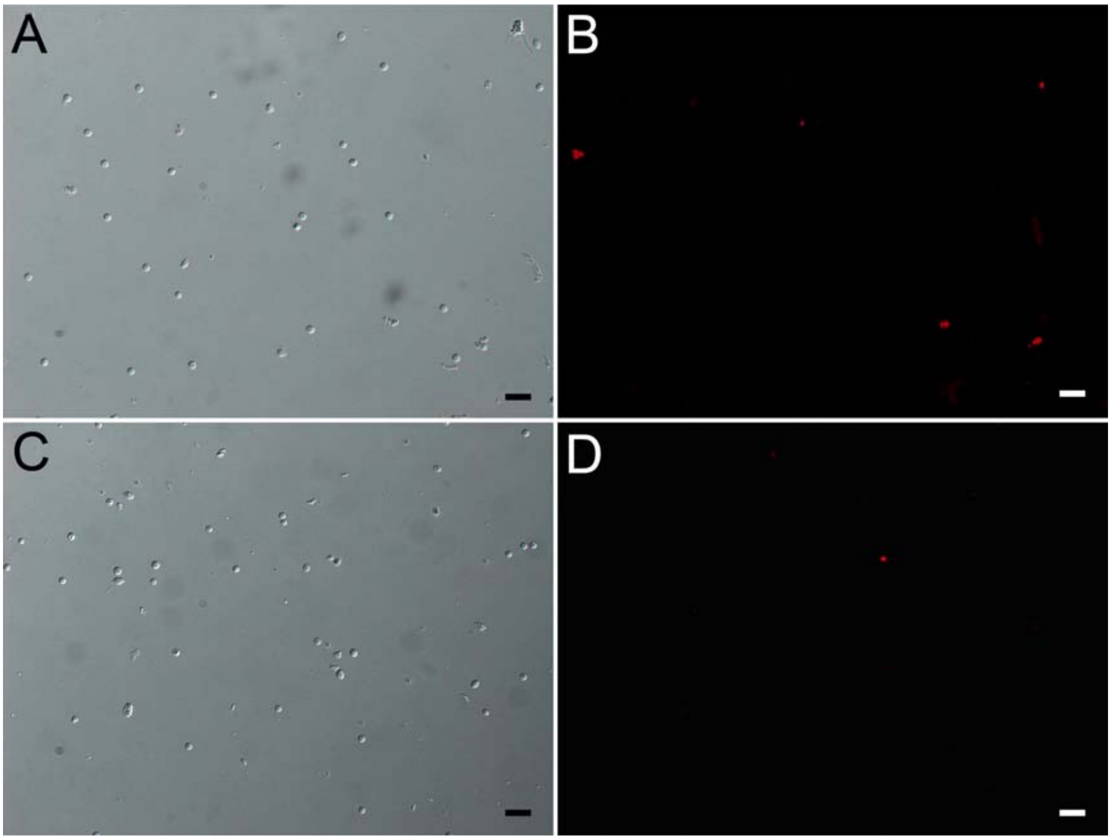

Figure S1. No vegetative nuclei detected in purified generative cells (GCs) and sperm cells (SCs) by propodium iodide **(PI) staining. (A, B)** Differential interference contrast (DIC) observation **(A)** and PI staining **(B)** of purified GCs. **(C, D)** DIC observation **(C)** and PI staining **(D)** of purified SCs. No VN except for a few debris possibly from ruptured GCs **(A, B)** or SCs **(C, D)**. Scale bar: 20 μm.

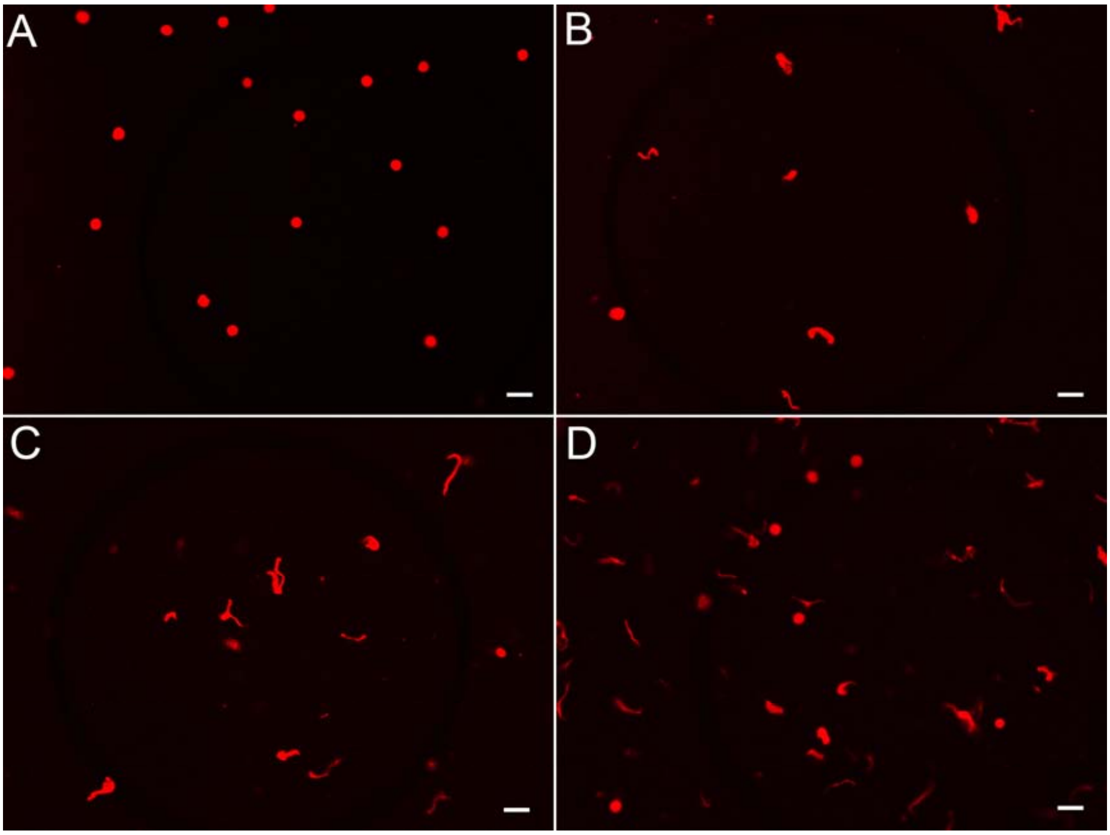

Figure S2. Effects of pipetting and washing on VN integrity. (A) VN isolated using Percoll gradient (A, control). (B, C) Pipetting through a 1000-μL tip with the top end cut off for 3 times (B) or 10 times (C). (D) Washing only once**.** The VN was stained by PI and observed under a microscope, showing that VN was impaired by pipetting and washing. Scale bar: 20 μm.

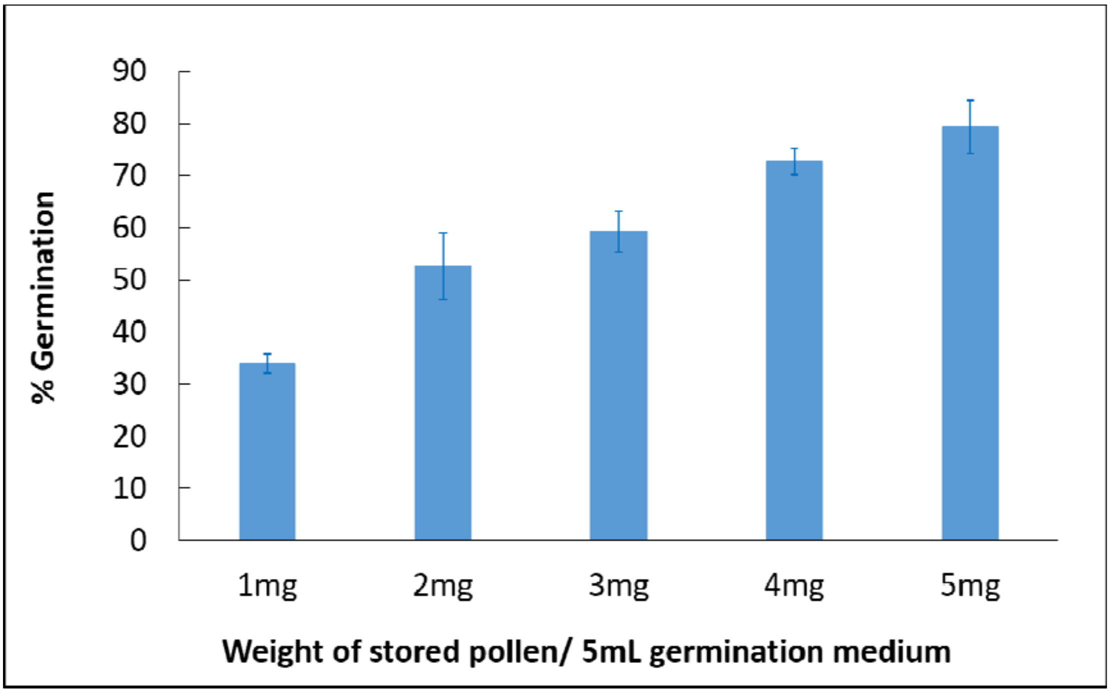

Figure S3. Effect of pollen density on germination rate *in vitro*. Data are mean±SD from 3 independent experiments.

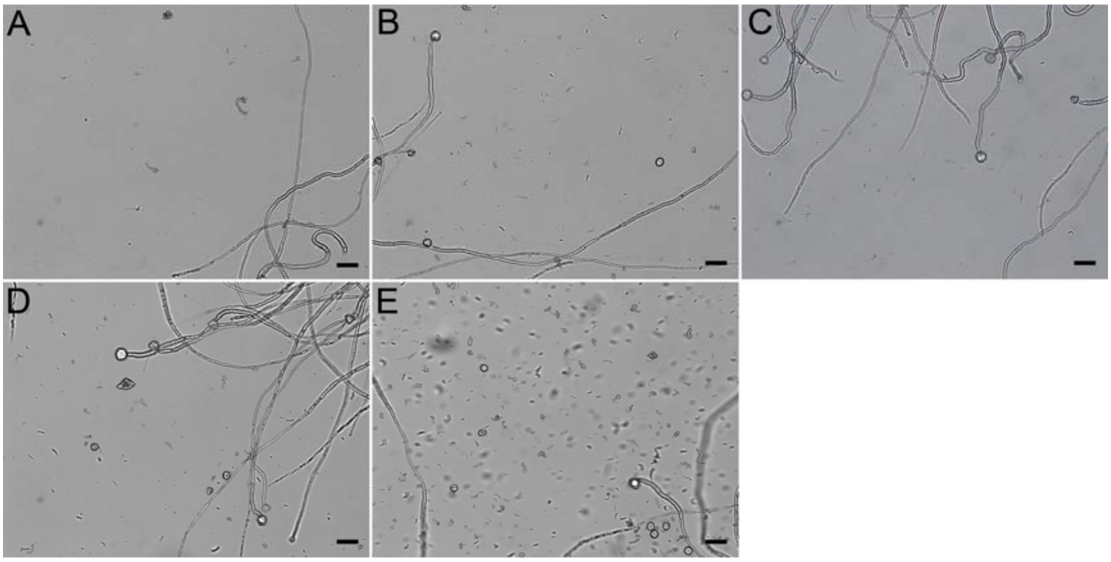

Figure S4. Different input of pollen grains affected the integrity of 10-h–cultured pollen tubes. A given amount (below) of pollen grains were pre-hydrated, then cultured in 5 mL germination medium for 10 h. The extent of pollen tube rupture was evaluated by observing the amount of cell debris in the medium. **(A)** 1 mg. **(B)** 2 mg. **(C)** 3 mg. **(D)** 4 mg. **(E)** 5 mg. Scale bar: 50μm.

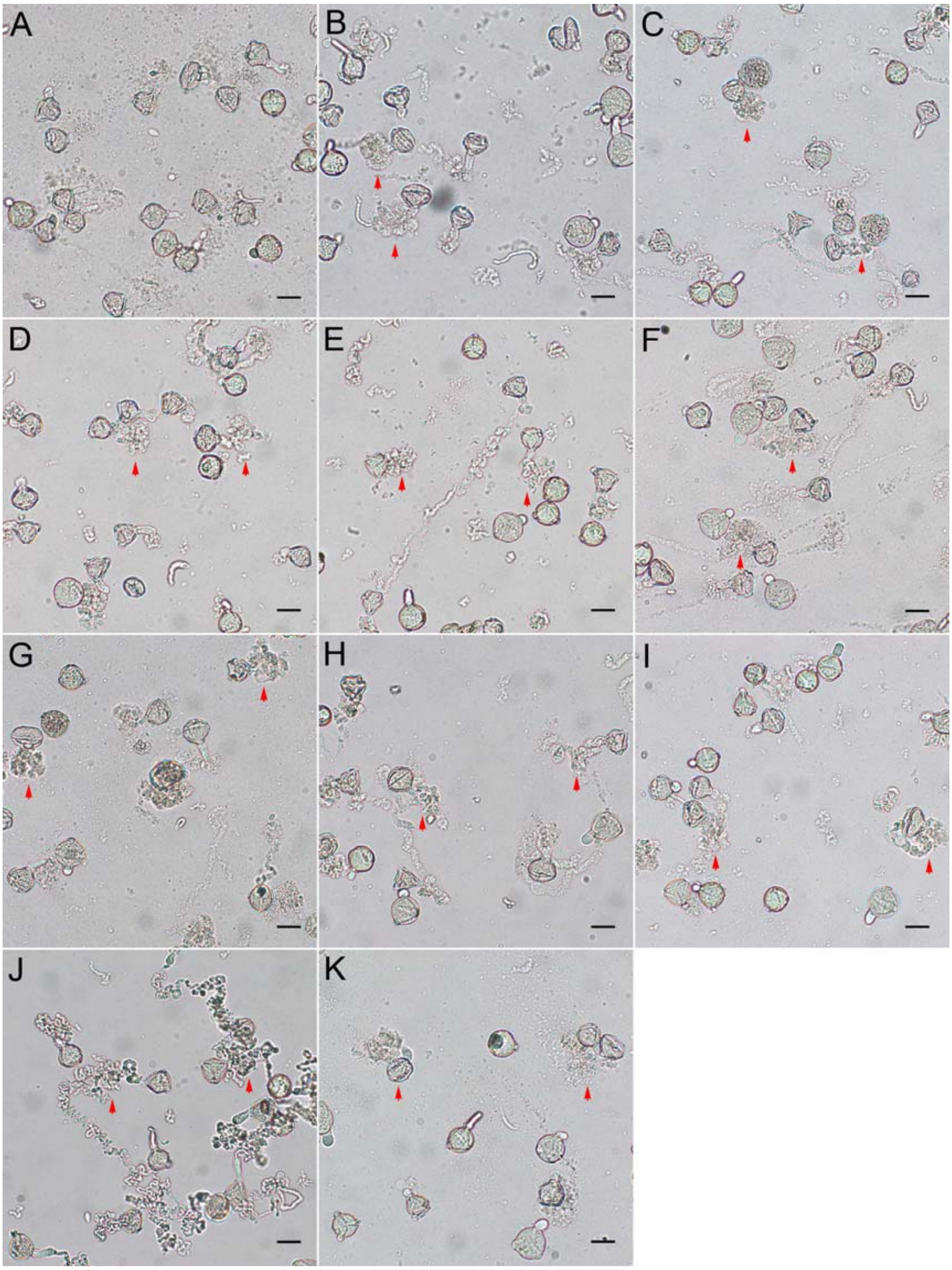

Figure S5. Components and pH value of osmotic shock solution affected the appearance of released cytoplasm from just-germinated pollen grains broken with osmotic shock solution during GC isolation. The released cytoplasm appeared not conglutinatous in solution with only 15.3% sucrose but appeared conglutinatous in solution containing MES or MOPS regardless of concentration. Acidic pH could

aggravate this appearance. **(A)** 15.3% sucrose. **(B)** 15.3% sucrose, 5 mM MES-KOH, pH6.0. **(C)** 15.3% sucrose, 10 mM MES-KOH, pH6.0. **(D)** 15.3% sucrose, 20 mM MES-KOH, pH6.0. **(E)** 15.3% sucrose, 40 mM MES-KOH, pH6.0. **(F)** 15.3% sucrose, 5 mM MOPS-KOH, pH6.0. **(G)** 15.3% sucrose, 10 mM MOPS-KOH, pH6.0. **(H)** 15.3% sucrose, 20 mM MOPS-KOH, pH6.0. **(I)** 15.3% sucrose, 40 mM MOPS-KOH, pH6.0. **(J)** 15.3% sucrose, 20 mM MES-HCl, pH5.0. **(K)** 15.3% sucrose, 20 mM MES-KOH, pH7.0. Arrow indicates conglutinated cytoplasm. Scale bar: 20 µm.

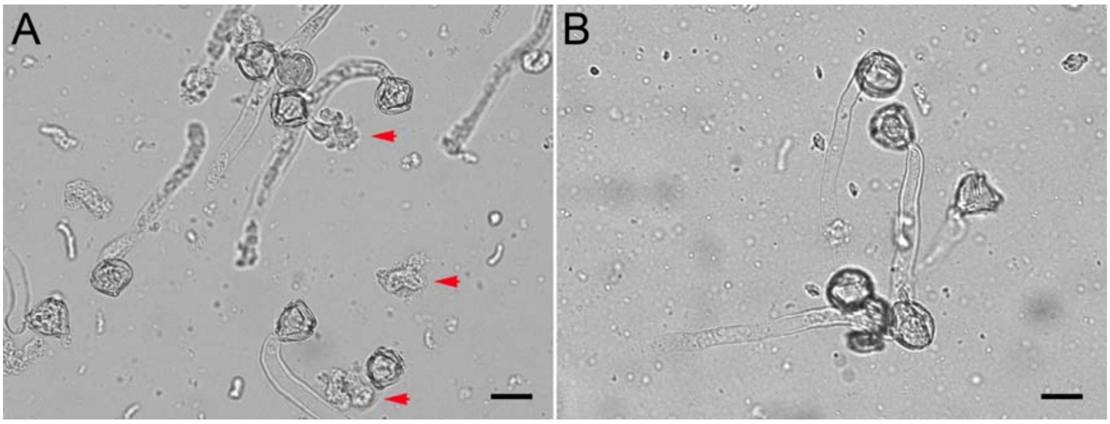

Figure S6. The pH value of enzymolysis solution used to release VN affected the appearance of the released cytoplasm. **(A)** 2.5% sucrose, 9.5% mannitol, 5 mM $EDTANa_2$, 10 mM MOPS, pH5.6. **(B)** 2.5% sucrose, 9.5% mannitol, 5 mM $EDTANa_2$, 10 mM MOPS-NaOH, pH7.2. Arrow indicates conglutinated cytoplasm. Scale bar: 20 µm.
